# Transcriptome analysis of monocyte-HIV interactions

**DOI:** 10.1186/1742-4690-7-53

**Published:** 2010-06-14

**Authors:** Rafael Van den Bergh, Eric Florence, Erika Vlieghe, Tom Boonefaes, Johan Grooten, Erica Houthuys, Huyen Thi Thanh Tran, Youssef Gali, Patrick De Baetselier, Guido Vanham, Geert Raes

**Affiliations:** 1 Department of Molecular and Cellular Interactions, VIB, Brussels, Belgium; 2 Laboratory of Cellular and Molecular Immunology, Vrije Universiteit Brussel, Brussels, Belgium; 3 HIV/STD Unit, Department of Clinical Sciences, Institute of Tropical Medicine, Antwerp, Belgium; 4 Laboratory of Molecular Immunology, Department of Biomedical Molecular Biology, Ghent University, Ghent, Belgium; 5 Unit of Molecular Pathophysiology and Experimental Therapy, Department for Molecular Biomedical Research, VIB, Ghent, Belgium; 6 Unit of Molecular Pathophysiology and Experimental Therapy, Department of Biomedical Molecular Biology, Ghent University, Ghent, Belgium; 7 HIV Virology Unit, Department of Microbiology, Institute of Tropical Medicine, Antwerp, Belgium; 8 Department of Biomedical Sciences, Faculty of Pharmaceutical, Biomedical and Veterinary Sciences, University of Antwerp, Antwerp, and Faculty of Medicine and Pharmacy, Vrije Universiteit Brussel, Brussels, Belgium

## Abstract

**Background:**

During HIV infection and/or antiretroviral therapy (ART), monocytes and macrophages exhibit a wide range of dysfunctions which contribute significantly to HIV pathogenesis and therapy-associated complications. Nevertheless, the molecular components which contribute to these dysfunctions remain elusive. We therefore applied a parallel approach of genome-wide microarray analysis and focused gene expression profiling on monocytes from patients in different stages of HIV infection and/or ART to further characterise these dysfunctions.

**Results:**

Processes involved in apoptosis, cell cycle, lipid metabolism, proteasome function, protein trafficking and transcriptional regulation were identified as areas of monocyte dysfunction during HIV infection. Individual genes potentially contributing to these monocyte dysfunctions included several novel factors. One of these is the adipocytokine NAMPT/visfatin, which we show to be capable of inhibiting HIV at an early step in its life cycle. Roughly half of all genes identified were restored to control levels under ART, while the others represented a persistent dysregulation. Additionally, several candidate biomarkers (in particular CCL1 and CYP2C19) for the development of the abacavir hypersensitivity reaction were suggested.

**Conclusions:**

Previously described areas of monocyte dysfunction during HIV infection were confirmed, and novel themes were identified. Furthermore, individual genes associated with these dysfunctions and with ART-associated disorders were pinpointed. These genes form a useful basis for further functional studies concerning the contribution of monocytes/macrophages to HIV pathogenesis. One such gene, NAMPT/visfatin, represents a possible novel restriction factor for HIV.

## Background

Both macrophages and T lymphocyte subsets express the CD4 receptor and either the CXCR4 and/or the CCR5 coreceptor which confer susceptibility to infection with the Human Immunodeficiency Virus (HIV). Upon infection, CD4^+ ^T lymphocytes typically succumb to the cytopathic effect of the virus [1], and the gradual depletion of the CD4^+ ^T lymphocyte pool has been considered a hallmark of HIV infection and the development of the Acquired Immune Deficiency Syndrome (AIDS) since the early days of the HIV pandemic. Macrophages, on the other hand, do not tend to suffer from the cytopathic effects mediated by the virus [2,3], but instead develop a wide array of dysfunctions which contribute significantly to the pathogenesis of HIV infection. Despite the recognition of macrophage contribution to HIV pathogenesis early on in HIV research [4,5], most studies have focused and continue to focus on T lymphocyte depletion and/or dysfunction, and many of the molecular mechanisms underlying the macrophage dysfunction during HIV infection remain poorly characterised. Nevertheless, as pointed out by other authors [6], in the combination Antiretroviral Therapy (ART) era where viral suppression in T lymphocytes is increasingly more efficient, the understanding of the viral mechanisms in other reservoir cells such as macrophages becomes ever more crucial.

Aberrant HIV-induced macrophage behaviour can be classified as relatively straightforward loss of function, such as reduced phagocytosis [7,8] and antigen presentation [9], or as more complex dysfunction. Such dysfunctions include a direct contribution to the establishment, spread and persistence of the infection: as long-living primary target cells of HIV with a wide-spread dissemination and a persistent failure to enter apoptosis upon infection [10,11], they represent an important cellular reservoir for the virus [12]. Additionally, macrophages exacerbate disease progression by contributing to T lymphocyte depletion: HIV infected macrophages have been documented to participate in the killing of uninfected CD4^+ ^and CD8^+ ^T lymphocytes, while at the same time protecting infected CD4^+ ^T lymphocytes from apoptosis [13]. Furthermore, infected and uninfected macrophages can contribute to sustained chronic immune activation during HIV infection, e.g. through the perturbation of cytokine and chemokine networks [14-16]. With the acknowledged notion of chronic immune activation as a paradoxical driving force of immune suppression [17], this pro-inflammatory macrophage phenotype during HIV infection may be a crucial parameter in disease progression. Yet other macrophage dysfunctions are associated with more peripheral HIV- or ART-associated disorders such as atherosclerosis [18], lipodystrophy [19], and metabolic syndrome during HIV infection and/or combination ART [20,21].

Monocytes, for their part, are much less permissive to infection with HIV, both *in vitro *[22] and *in vivo*, where estimates of infected circulating monocytes are consistently low [23,24]. Circulating monocytes represent the most accessible primary model for macrophage dysfunction during HIV infection, however, and are furthermore of sufficient importance to study in their own right. Infectious virus can be recovered from circulating monocytes, both in untreated patients [24] and in patients undergoing long-term successful combination ART [25]. Additionally, the circulating monocyte pool as a whole does seem to be affected during HIV infection, despite the low frequency of actually infected monocytes. Transcriptome studies, in particular, show a form of hybrid phenotype exhibiting both increased and decreased pro-inflammatory features [26,27]. This modulation of the non-infected monocyte population could be due to the virus itself through mechanisms which do not require direct infection [28], or to other factors contributing to (aberrant) immune activation occurring during HIV infection, such as perturbed cytokine networks [29] or other inflammatory stimulants [30].

Several key factors in the described dysregulated processes have been identified [18,31], but many molecular components remain elusive. Furthermore, other aspects of HIV and combination ART pathogenesis in which monocyte/macrophage dysfunction is involved may only now be emerging or remain yet to be discovered, in particular in view of the limited number of studies focussing on the monocyte response to ART [32]. In order to generate novel hypotheses rather than test pre-existing ones in the context of monocyte-HIV interactions, we performed a transcriptome analysis on monocyte samples from patients in different stages of HIV infection and/or combination ART treatment, using a parallel approach of genome-wide microarray analysis and focused gene expression profiling to identify broad areas of monocyte dysfunction and to pinpoint genes which are potentially involved in one or several of these dysfunctions. In particular the factors which are exploited by the monocyte/macrophage to communicate with and/or modulate other immune cells were of interest, as they represent a particularly relevant population [33,34] which is a primary target for intervention.

## Methods

### Sample collection

For the cross-sectional study on the effects of HIV infection, 50 ml blood samples were collected in EDTA-tubes from therapy-naïve HIV-1-seropositive patients from the HIV-Clinic of the Institute of Tropical Medicine in Antwerp, Belgium (inclusion of all therapy-naïve seropositive patients, irrespective of viral load (VL) and/or CD4^+ ^T lymphocyte (CD4T) count; n = 29). For the longitudinal study on the effects of combination ART, 20 ml blood samples were collected in EDTA-tubes from therapy-naïve patients at baseline and at 3, 6 and 9 months after therapy initiation (NRTI+PI regimen only). In all patients but one the indication for ART was a decline in CD4T ≤ 350 cell/mm>^3^; irrespective of VL (n = 16). As controls, 50 ml blood samples were collected in EDTA-tubes from self-asserted HIV seronegative blood donors without apparent infections, in the same age range as the HIV patients (n = 15). The study was approved by the Institutional Review Board of the Institute of Tropical Medicine, and written informed consent was obtained from all donors. Patient characteristics are shown in table 1 (cross-sectional) and table 2 (longitudinal).

**Table 1:** Clinical information of therapy-naïve HIV-1 seropositive donors (cross-sectional study)

Patient ID	Experiment	CD4T count (cells/mm^3^)	VL (log copies/ml)	Patient ID	Experiment	CD4T count (cells/mm^3^)	VL (log copies/ml)
TN 01	MAS & CL	133	2.70	TN 16	MAS	503	4.32
TN 02	MAS & CL	142	2.28	TN 17	MAS	532	4.78
TN 03	MAS & CL	197	5.91	TN 18	MAS	535	4.78
TN 04	MAS	226	5.59	TN 19	MAS	540	4.36
TN 05	MAS	233	5.59	TN 20	MAS & CL	644	4.34
TN 06	MAS	311	4.97	TN 21	MAS	738	5.58
TN 07	MAS	329	5.37	TN 22	MAS	746	4.90
TN 08	MAS & CL	359	3.87	TN 23	MAS & CL	748	5.54
TN 09	MAS	359	5.84	TN 24	MAS	756	5.07
TN 10	MAS	371	3.60	TN 25	MAS	760	3.93
TN 11	MAS	374	4.24	TN 26	MAS	778	5.00
TN 12	MAS	382	4.00	TN 27	MAS	781	3.50
TN 13	MAS	436	4.28	TN 28	MAS & CL	856	4.82
TN 14	MAS & CL	446	3.91	TN 29	MAS	1026	3.08
TN 15	MAS	462	4.06				

**Table 2:** Clinical information of HIV-1 seropositive donors on combination ART (longitudinal study)

		CD4T count (cells/mm^3^)				VL (log copies/ml)			
Patient ID	Experiment	*BL*	*M3*	*M6*	*M9*	*BL*	*M3*	*M6*	*M9*
HA 01	MAS	239	373	407	502	4.61	2.37	< 1.70	< 1.70
HA 02	MAS	153	222	353	263	5.36	1.75	1.85	< 1.70
HA 03	MAS	193	441	446	437	5.36	2.84	1.72	2.05
HA 04	MAS	273	608	577	761	4.58	< 1.70	< 1.70	< 1.70
HA 05	MAS	548	592	956	778	4.88	2.12	< 1.70	< 1.70
HA 06	MAS	239	317	348	591	5.01	< 2.60	< 1.70	< 1.70
HA 07	MAS	165	209	282	222	5.13	< 2.60	< 2.60	< 1.70
HA 08	MAS	146	241	264	315	4.48	< 1.70	< 1.70	< 1.70
HA 09	MAS	205	ND	400	318	5.45	ND	< 1.70	< 1.70
HA 10	MAS	269	327	451	372	5.26	< 1.70	< 1.70	< 1.70
HA 11	MAS	324	707	561	590	5.68	3.26	< 2.60	< 2.60
HA 12	MAS	202	245	254	242	5.16	< 1.70	< 1.70	< 1.70
HA 13	MAS	261	ND	425	432	5.77	ND	< 1.70	< 1.70
HA 14	MAS	318	257	270	338	5.14	< 1.70	< 1.70	< 1.70
HA 15	MAS	258	524	462	300	4.57	2.35	< 1.70	< 1.70
HA 16	MAS	232	356	358	318	5.57	< 2.60	3.85	< 1.70

MAS: custom Macrophage Activation State array platform; CL: commercial CodeLink HWG bioarray platform; CD4T: CD4^+ ^T lymphocyte; VL: viral load; BL: baseline; M3/6/9: sample taken resp. 3, 6 and 9 months after therapy initiation; ND: not done.

Peripheral blood mononuclear cells (PBMC's) were separated by Lymphoprep (Axis Shield, Dundee, United Kingdom) gradient. Monocytes were purified from the PBMC fraction using the negative selection-based Monocyte Isolation Kit II from Miltenyi-Biotec (Bergisch Gladbach, Germany), according to the manufacturer's instructions. Yields were minimally 5 million monocytes with a purity > 85%, as verified through flow cytometry. For RNA extraction, monocytes were immediately lysed in Trizol (Invitrogen, Carlsbad, CA, USA) and lysates were stored at -80°C.

### RNA and protein isolation

Total RNA was prepared from Trizol lysates by chloroform extraction, as per the manufacturer's recommendations. Ten randomly selected samples were checked for integrity on a BioAnalyzer (BioRad, Hercules, CA, USA): no contamination or degradation of RNA was detected. Subsequently, the protein fraction was purified from the Trizol pellets by isopropanol precipitation, again according to the manufacturer's instructions.

### CodeLink arrays

Selected RNA samples were prepared and hybridised to CodeLink HWG bioarrays (Amersham Biosciences, Freiberg, Germany; now Applied Microarrays, Tempe, AZ, USA - http://www.appliedmicroarrays.com) by the VIB MicroArray Facility http://www.microarrays.be. Total RNA was controlled for integrity and purity using an Agilent Bioanalyzer and a NanoDrop spectrophotometer, respectively. All samples were of similar RNA quality. Starting with 1 μg of total RNA, the RNA amplification was performed by in vitro transcription (IVT) with a biotin labeling reaction during the IVT, according to the recommendations of the manufacturer (Amersham Biosciences). A set of bacterial control mRNAs was added to the RNA as controls for the IVT reaction. The probes were purified and analyzed again for yield (> 20 μg) and purity (260:280 nm and 260:230 nm > 1.8). 10 μg of the resulting antisense RNA was fragmented according to the recommendations of the manufacturer (Amersham Biosciences) and resuspended in 260 μl of hybridization buffer.

The gene array chips were hybridized in a shaker-incubator at 37°C at 300 rpm for 18 hours and washed and stained with Cy5-Streptavidin according to the recommendations of the manufacturer (Amersham Biosciences). The DNA Microarray scanner of Agilent was used for scanning and image analysis was performed with the Codelink Expression Analysis 4.1 software. Datasets were deposited at the EMBL-EBI repository (accession E-MEXP-2255).

### Macrophage Activation State arrays

The Macrophage Activation State (MAS) array was developed as a focused and flexible tool for the analysis of gene expression patterns in monocytes/macrophages (manuscript in preparation). A collection of genes (ca. 700) associated with different macrophage activation states was compiled, using a combination of literature data-mining and human 'translation' of murine models of macrophage activation available in our laboratory (the complete gene population represented on this array is documented in Additional file 1). Subsequently, gene specific primers were designed for the genes in this collection and fragments were amplified from total cDNA pools of monocytes under various *in vitro *and *in vivo *conditions. These fragments were applied in duplicate on 7 × 10 cm nylon membranes and were cross-linked to the membranes using UV-exposure.

RNA samples from all patients were selected for analysis on this MAS array. A reverse transcription was performed on 1 μg total RNA using oligo-dT and Superscript II reverse transcriptase (Invitrogen) in the presence of ^33^P-dCTP (Amersham Biosciences), and the labelled cDNA was then hybridised to the membranes for 20 h at 42°C in NorthernMax hybridisation buffer (Ambion, Austin, TX, USA). Membranes were subsequently washed with SDS-containing buffer at 68°C and were exposed to a phosphorscreen to reveal bound radioactivity. Phosphorscreens were then scanned in a phospho-imager (BioRad). Spot recognition and quantification, background correction and array normalisation were performed using custom-designed software based on the program ImageJ (Image Processing and Analysis in Java, Sun Microsystems, Santa Clara, CA, USA).

### Real-time semi-quantitative PCR

mRNA expression of the individual genes of interest was examined using real-time semi-quantitative PCR (RT-qPCR). cDNA was prepared from 1 μg total RNA using oligo-dT and Superscript II reverse transcriptase (Invitrogen). Gene specific primers for the genes of interest and the housekeeping gene GAPDH (Entrez GeneID: 2597) were used to run PCR reactions in duplicate in a BioRad MyCycler, with BioRad iQ SYBR Green Supermix. Gene expression was normalised using GAPDH as a housekeeping gene. Sequences of the gene specific primers are supplied as Additional file 2.

### *In vitro *infection experiments

For *in vitro *infection experiments, PBMC's were separated by Lymphoprep (Axis Shield, Dundee, United Kingdom) gradient from buffy coats of healthy donors of the Blood

Transfusion Centre of Antwerp and were either employed as such in PBMC infection experiments or were used for monocyte preparation. Monocytes were purified from PBMC by magnetic isolation using CD14 microbeads (Miltenyi-Biotec) according to the manufacturer's instructions. Yields were minimally 50 million monocytes with a purity > 98%, as verified through flow cytometry. These cells were then differentiated to monocyte-derived macrophages (MDM) during 7 days in RPMI 1640 medium (Bio-Whittaker, Verviers, Belgium) supplemented with 10% bovine fetal calf serum (Biochrom, Berlin, Germany), penicillin (100 U/ml) and streptomycin (100 μg/ml) (Roche) and 40 ng/ml M-CSF (PeproTech, London, United Kingdom) at 37°C and 5.0% CO_2_. Half of the medium was replaced after 4 days of culture. Cells were harvested and used for experiments in the same medium (without M-CSF). All experiments were repeated with cells from three independent donors.

Virus stocks (HIV_BaL_, HIV_968-2 _and HIV_968-3_) were prepared by short-term propagation in PHA/IL2-stimulated PBMC from HIV seronegative donors as described previously [35].

Recombinant factors (CCL2, NAMPT and PDGFC) were obtained from PeproTech; viability of cells treated with the recombinant factors was evaluated using the cell proliferation agent WST-1 (Roche) according to the manufacturer's instructions: no appreciable effect on cell viability was observed at the concentrations used (data not shown). For infections, MDM or non-activated PBMC were plated in 96-well plates at 7.5 × 10^5 ^cells/ml and pre-treated with recombinant CCL2 (20 ng/ml), NAMPT (100 ng/ml) or PDGFC (20 ng/ml) for 24 hours at 37°C and 5.0% CO_2_. Then, a dilution series of virus was added in sixfold and incubated for 24 hours, again at 37°C and 5.0% CO_2_. Cells were then washed 3 × to remove unbound virus and incubated for 14 days in the presence of 5 ng/ml IL2 (Roche) and 0.5 μg/ml phytohemagglutinin (PHA; Murex Biotech Ltd., Dartford, United Kingdom) for PBMC and in complete medium without cytokines for macrophages. Productive infection was monitored via an in-house developed p24 antigen ELISA, as described elsewhere [35]. Viral infectivity was quantified as the TCID50 (50% tissue culture infectious dose) value, which was calculated by the method of Reed & Muench [36]. For viral binding experiments, the same procedure was followed (pre-incubation with NAMPT of 4 hours instead of 24 hours), but cells were incubated with the virus for 2 hours and were then lysed in 200 μl NP40 solution after washing. p24 content of the lysate was then assessed by ELISA to quantify the bound virus.

For proviral quantification experiments, MDM or non-activated PBMC were plated in 24-well plates at 1 × 10^6 ^cells/ml and pre-treated with recombinant visfatin (200 ng/ml) for 24 hours at 37°C and 5.0% CO_2_. Then, virus was added at a multiplicity of infection of 0.1 and 0.001 and incubated for 24, again at 37°C and 5.0% CO_2_. Cells were then immediately lysed in Trizol (Invitrogen) and genomic DNA was prepared from the Trizol pellets as per the manufacturer's recommendations. Proviral DNA levels were determined semi-quantitatively by RT-qPCR: gene specific primers for the viral LTR region (LTR_NEC152: 5'-GCCTCAATAAAGCTTGCCTTGA-3' and LTR_NEC131: 5'-GGCGCCACTGCTAGAGATTTT-3') and the genomic housekeeping fragment ERV-3 (PHP10-F: 5'-CATGGGAAGCAAGGGAACTAATG-3' and PHP10-R: 5'-CCCAGCGAGCAATACAGAATTT-3') were used to run PCR reactions in duplicate in a BioRad MyCycler, with BioRad iQ SYBR Green Supermix. Proviral DNA was normalised using ERV-3 as a housekeeping gene, as discussed elsewhere [37].

### Nampt-Elisa

An ELISA kit for NAMPT/visfatin (AdipoGen, Seoul, Korea) was used for NAMPT detection, as suggested by Körner and colleagues [38]. Plasma samples (undiluted) of HIV patients and healthy control donors were analysed according to the manufacturer's instructions.

### NAMPT-Western Blot

Total cellular NAMPT was detected by Enhanced Chemoluminescence (ECL) Western Blot. 30 μg samples were run on a 10% SDS-PAGE gel and transferred to PVDF membranes using the iBlot Dry Blotting System (Invitrogen) according to the manufacturer's instructions. A rabbit anti-NAMPT polyclonal Ab (Bethyl Laboratories, Montgomery, TX, US) at 1:3000 dilution and an an anti-rabbit-HRP conjugate (Sigma-Aldrich, Saint Louis, MO, US) at 1:10000 dilution were used to probe these membranes. The membranes were subsequently incubated for 5 minutes with SuperSignal West Pico Chemiluminescent Substrate (Pierce, Rockford, IL, US) and exposed to photosensitive film. Films were developed using a Fujifilm FPM-100A developer (Fujifilm, Tokyo, Japan). After exposure, the membranes were incubated in 50% H_2_O_2 _to saturate the bound HRP and were reprobed in the same fashion for the housekeeping protein β-actin.

### *In vitro *assessment of NAMPT activity

MDM generated as described above, plated in 96-well plates at 7.5 × 10^5 ^cells/ml, were stimulated with NAMPT (200 ng/ml) and *E. coli *lipopolysaccharide (LPS) (100 ng/ml) for 2 days. Secretion of the β-chemokines MIP1α (CCL3), MIP1β (CCL4) and RANTES (CCL5) was assessed by Cytometric Bead Assay (CBA) (Becton Dickinson, Erembodegem, Belgium) in cell culture supernatants according to the manufacturer's instructions. Additionally, CCR5 and CXCR4 expression on stimulated MDM was assessed in flow cytometry as described previously [39].

### Statistical analysis

All microarray datasets were processed using the GeneMaths XT software package (Applied Maths, St.-Martens-Latem, Belgium).

For CodeLink HWG bioarrays, all genes were re-annotated (i.e. updating of replaced Gene ID's, etc.) using the 22.01.2009 releases of the Entrez and UniGene databases. A dataset was compiled after background correction (subtract algorithm) and array normalisation (mean algorithm). A set of differentially expressed genes was compiled by filtering the data according to three criteria: (1) **statistical significance**: *p*-value as determined by Student's *t *test < 0.01 (or for a more stringent analysis: *p*-value after Benjamini-Hochberg correction [40] for FDR control < 0.1); (2) **reliability**: a spot quality flag G ("good", a quality flag assigned by the CodeLink software package) in all arrays and (3) **relevance**: a fold change between the means of the two groups ≥ 1.5.

Overrepresentation analysis was performed on processed CodeLink datasets using the application Gene Map Annotator and Pathway Profiler (GenMAPP) [41] v.2.1 and the associated program MAPPFinder [42] v.2 (based on the Gene Ontology (GO) annotations provided by the GO Consortium[43]). Pathways which were identified by these software packages were subjected to filtering criteria: (1) number of "changed" (i.e. significantly differentially expressed) genes in a pathway ≥ 3; (2) z-score ≥ 1.96 and (3) permute p-value ≤ 0.05.

For MAS arrays, datasets were compiled as mentioned above. Sets of differentially expressed genes were compiled by filtering the data according to (1) **statistical significance**: *p*-value as determined by an uncorrected Mann-Whitney test < 0.05 (for the cross-sectional study) or a *p*-value < 0.05 in ANOVA (for the longitudinal study); (2) **reliability**: variation between spot replicates ≤ 20% and (3) **relevance**: a fold change between the means of two groups (HIV versus controls or ART baseline versus ART timepoints) ≥ 1.5. The error rate was estimated by RT-qPCR and training/comparison set validation, using the cross-sectional study as training set and the baseline samples of the longitudinal study as comparison set. For the smaller population sizes in the analysis of genes associated with the abacavir hypersensitivity reaction, an uncorrected Student's t-test and a more stringent fold change cut-off of 2.5 were used to identify differentially expressed genes.

Correlation of gene expression with viral load and/or CD4T count was assessed via Spearman correlation test. All viral infection data are expressed as mean ± SEM; representative data of at least three independent experiments are shown, except where indicated. NAMPT expression data and plasma loads were assessed by nonparametric Mann-Whitney test.

## Results

### Identification of perturbed gene networks in monocytes of therapy-naïve HIV patients

To identify the areas of monocyte dysfunction in our patient population, eight therapy-naïve HIV patient samples and four healthy control samples (table 1) were selected for analysis on CodeLink HWG microarrays. Samples with a broad range of CD4T counts representative for the full patient population, and healthy controls in the same age range, were chosen. While the sample number in this preliminary experiment was too low to identify reliable individual biomarkers with sufficient statistical power, these datasets can be used to distinguish the broad cellular processes or pathways which are modulated as a whole by HIV infection. Samples were grouped according to HIV sero-status, i.e. no stratifications according to CD4T count or viral load were performed. A collection of 91 differentially expressed genes (172 using the less stringent conditions) was compiled (supplied as Additional file 3).

The processed datasets were then analysed using GenMAPP and MAPPFinder to identify the global biological trends in our expression data. This over representation analysis revealed a set of processes which appear to be modulated/dysregulated to a significant degree in monocytes of therapy-naïve HIV patients (table 3). The most specific GO term which is still significant is shown: i.e. when for example "regulation of transcription" and its daughter term "negative regulation of transcription" are called as significant, we show only this second term. Several of these processes, such as transcriptional regulation and cell cycle modulation, were previously identified in other transcriptome studies as modulated by HIV in monocytes and monocyte-derived macrophages (MDM) (reviewed in e.g. [32]). Others, such as lipid metabolism and proteasome function, have been linked with HIV-monocyte/macrophage interactions [18,44], but were to the best of our knowledge not yet described in the context of a transcriptome analysis. Establishment of these broad areas of gene dysregulation in monocytes during HIV infection allowed us to classify genes in our subsequent analyses.

**Table 3:** Overrepresentation of biological processes in differential gene expression data of monocytes from therapy-naïve HIV patients

**Class**	**GO term**	**GO ID**^1^	**z-score**^2^	***p*-value**^3^
**Apoptosis/DNA damage**				
	induction of apoptosis	6917	2.622	0.03
	response to radiation	9314	3.198	0.017
**Cell cycle**				
	cell maturation	48469	3.862	0.006
	positive regulation of cell proliferation	8284	3.062	0.009
**Lipid Metabolism**				
	Hs_Adipogenesis	User	2.896	0.018
**Proteasome activity**				
	**cysteine-type endopeptidase activity**	**4197**	**4.486**	**0.009**
	**Proteolysis**	**6508**	**1.996**	**0.044**
	**ubiquitin cycle**	**6512**	**2.271**	**0.041**
	**ubiquitin-protein ligase activity**	**4842**	**2.527**	**0.05**
**Protein trafficking**				
	**protein import into nucleus**	**6606**	**4.732**	**0.001**
**Transcriptional regulation**				
	**DNA binding**	**3677**	**2.929**	**0.003**
	**negative regulation of transcription**	**16481**	**3.207**	**0.012**
	**transcription regulator activity**	**30528**	**2.534**	**0.017**
	negative regulation of transcription\, DNA-dependent	45892	2.422	0.038

GO: Gene Ontology; ^1^: the official GO identification code for the process http://www.geneontology.org, "User" denotes a user contributed pathway; ^2^: the score for the standard statistical test under the hypergeometrical distribution, as calculated by MAPPFinder; ^3^: the permute *p*-value as correction on the z-score, as calculated by MAPPFinder; GO terms shown in **boldface **were identified using both the stringent and less stringent criteria, other terms were only found using the less stringent criteria.

### Focused transcriptome profiling of monocytes of therapy-naïve and combination ART-treated HIV patients

In parallel with this pathway-finding approach, we attempted to identify individual differentially expressed genes in a cross-sectional study of therapy-naïve HIV patients and in a longitudinal study of HIV patients on combination ART (table 1&2) using our custom MAS array platform. The cross-sectional group of therapy-naïve HIV patients was used as a training set: the gene expression in this population (n = 29) was compared with the expression in healthy control samples (n = 15) in order to identify genes with a differential expression between the groups, using our filtering criteria described above (significance/reliability/relevance). Furthermore, subgroups of patients with a high plasma viral load (VL ≥ 5 log copies/ml, n = 9) and/or a low CD4 T count (CD4 T ≤ 400 cells/mm^3^, n = 12) only were also compared with healthy control samples. The genes passing our selection (31 transcripts or 30 genes) were validated by RT-qPCR. Gene normalisation was performed using GAPDH expression. While several studies have suggested that GAPDH is suboptimal as a housekeeping gene in specific models (e.g. [45]) and that the enzymatic pathway in which it is involved may be modulated by HIV infection [46], our own analyses on several archetypal housekeeping genes indicated that GAPDH was stably expressed across all samples (not shown). In this way, we were able to compile a collection of genes (29 transcripts, 28 genes) for which the expression was associated with HIV serostatus in the training set of therapy-naïve samples.

**Figure 1:**
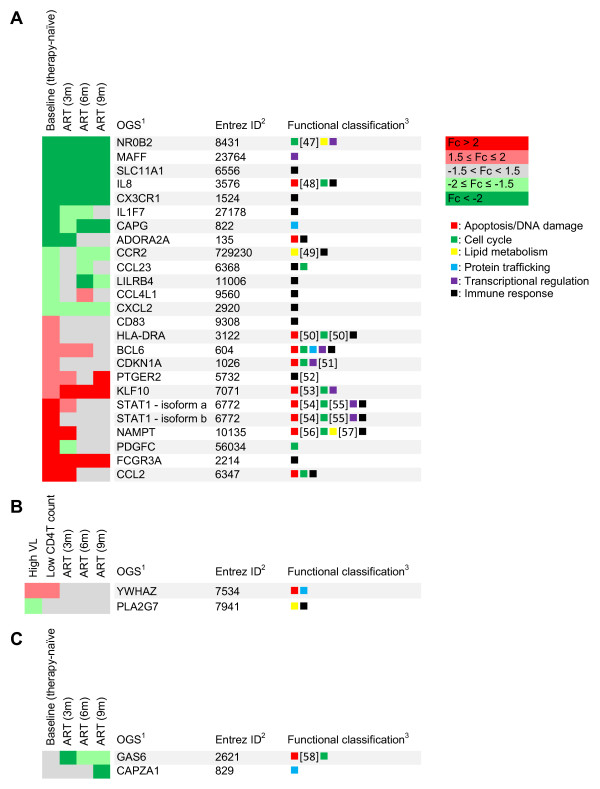
**Genes identified by transcriptome analysis in monocytes of HIV patients versus healthy controls**. A) Genes passing RT-qPCR and training/comparison set validation; mean fold change between the comparison set and healthy controls as assessed by RT-qPCR is shown at baseline and at 3, 6 and 9 months of therapy.^1^: the Official Gene Symbol (OGS, Entrez Gene); ^2^: the Entrez Gene identification code;^3^: Classification system (evidence for these classifications was derived from the Gene Ontology annotations, except where indicated); B) Genes identified only in patients with CD4T ≤ 400 cells/mm^3 ^and/or VL ≥ 5 log copies/ml; mean fold change between the comparison set and healthy controls as assessed by RT-qPCR is shown at baseline and at 3, 6 and 9 months of therapy. C) Genes identified by ANOVA as differentially regulated during therapy; mean fold change between the comparison set and healthy controls as assessed by RT-qPCR is shown at baseline and at 3, 6 and 9 months of therapy.

This collection of genes was then validated against the comparison set of baseline samples of the longitudinal study, which were analysed in the same fashion (MAS array profiling followed by RT-qPCR confirmation). 26 transcripts (25 genes) passed this validation (Figure 1A, references [47-57]), while 6 additional genes were found in the comparison set which were not identified in the training set. Furthermore, in the training set two additional genes were identified only in patients with high VL and/or low CD4T count (Figure 1B), while in the comparison set two genes modulated exclusively by therapy were identified by ANOVA (Figure 1C, reference [58]). For 14 of these transcripts expression was restored to control levels, while for 12 the expression remained dysregulated after 9 months of combination ART. An overview of the different classes of genes is presented in table 4.

**Table 4:** Classification of differentially expressed genes in monocytes of therapy-naïve and combination ART-treated HIV patients

	Modulated by HIV, persistent		Modulated by HIV, reversible		Modulated by ART	
	**OGS**^1^	**Entrez ID**^2^	**OGS**^1^	**Entrez ID**^2^	**OGS**^1^	**Entrez ID**^2^
**Down**	NR0B2	8431	**IL1F7**	**27178**	**GAS6**	**2621**
	MAFF	23764	ADORA2A	135	CAPZA1	829
	SLC11A1	6556	**CCL23**	**6368**		
	**IL8**	**3576**	**CCL4L1**	**9560**		
	CX3CR1	1524				
	CAPG	822				
	CCR2	729230				
	LILRB4	11006				
	**CXCL2**	**2920**				
**Up**	PTGER2	5732	CD83	9308		
	KLF10	7071	HLA-DRA	3122		
	FCGR3A	2214	BCL6	604		
			CDKN1A	1026		
			MARCKS	4082		
			STAT1 isoform - a	6772		
			STAT1 isoform - b	6772		
			**NAMPT**	**10135**		
			**PDGFC**	**56034**		
			**CCL2**	**6347**		

^1^: the Official Gene Symbol (OGS, Entrez Gene); ^2^: the Entrez Gene identification code. Genes encoding secreted factors are shown in **boldface**.

### Identification of genes associated with the abacavir hypersensitivity reaction

In the longitudinal arm of this study, we observed a hypersensitivity reaction to the drug abacavir in two out of seven patients, at the time unscreened for HLA-B*5701, who were receiving abacavir as a component of their combination ART regimen. Using our MAS array dataset, we compared monocyte gene expression patterns at baseline between patients with the hypersensitivity reaction and patients on the same regimen without adverse effects. We identified 6 genes which appear to be differentially expressed between patients who develop the abacavir hypersensitivity reaction and patients who do not: the cytoplasmic enzymes CA2 and CYP2C19, the chemokine CCL1, the transcription factor NFIB, and the transmembrane receptor NRP2 were upregulated in these patients, while the uncharacterised nuclear factor ANP32E was downregulated (Additional file 4). While these results lack statistical power due to the small population sizes, they are indicative of trends which may be of particular interest in the context of monocyte involvement in the hypersensitivity reaction or in the pursuit of biomarkers with diagnostic or prognostic value.

### Upregulation of an innate immune factor with inhibitory capacities against HIV

**Figure 2:**
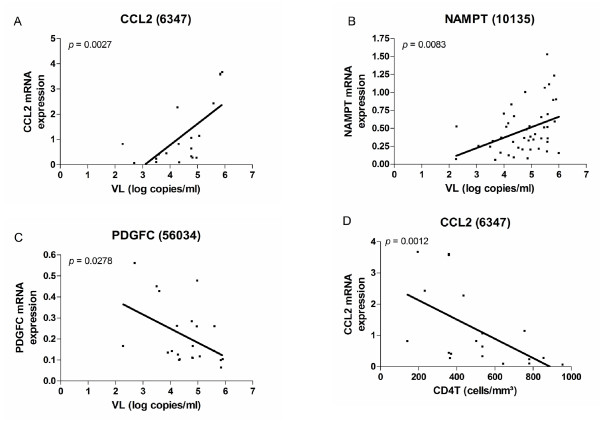
**A-C) Correlation of mRNA gene expression in monocytes of therapy-naïve HIV patients, as assessed by RT-qPCR, with the viral load; D) Correlation of mRNA gene expression in monocytes of therapy-naïve HIV patients, as assessed by RT-qPCR, with the CD4^+ ^T lymphocyte count - *p*-values for Spearman correlation testing are shown**.

**Figure 3:**
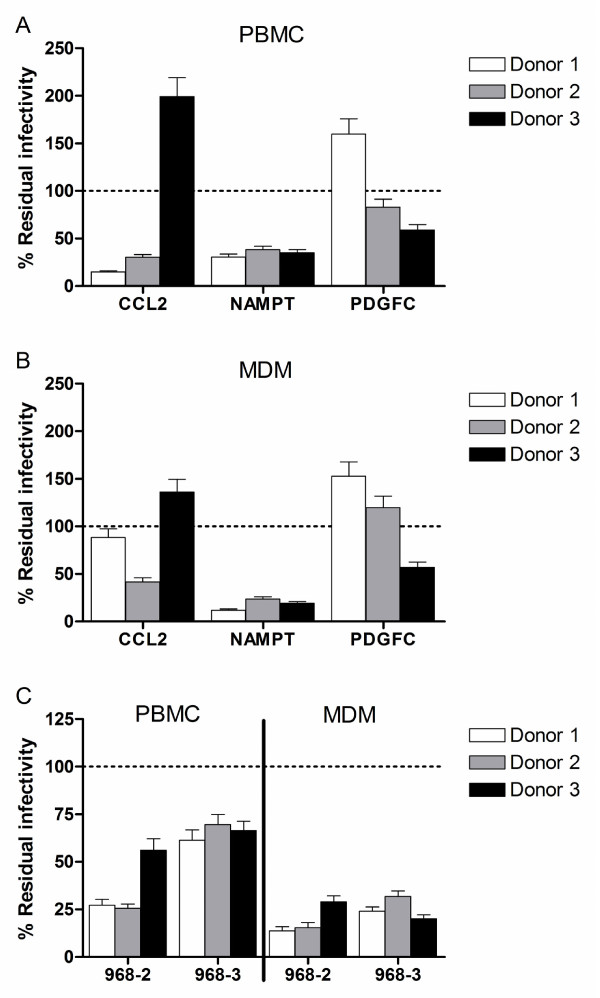
**Modulation of viral infectivity of the lab-attenuated strain HIV_BaL _by the secreted factors CCL2, NAMPT and PDGFC: infection of A) PBMC and B) MDM (pre-)treated with recombinant factors by HIV_BaL_**. C) Modulation of the viral infectivity of the biological clones HIV_968-2 _and HIV_968-3 _by the secreted factor NAMPT in PBMC and MDM. TCID50 values were determined using the method of Reed & Muench[36], based on p24 measurement in culture supernatants. Infectivity in treated cells is expressed as a percentage of infectivity in untreated control cells. Results in 3 independent donors are shown.

**Figure 4:**
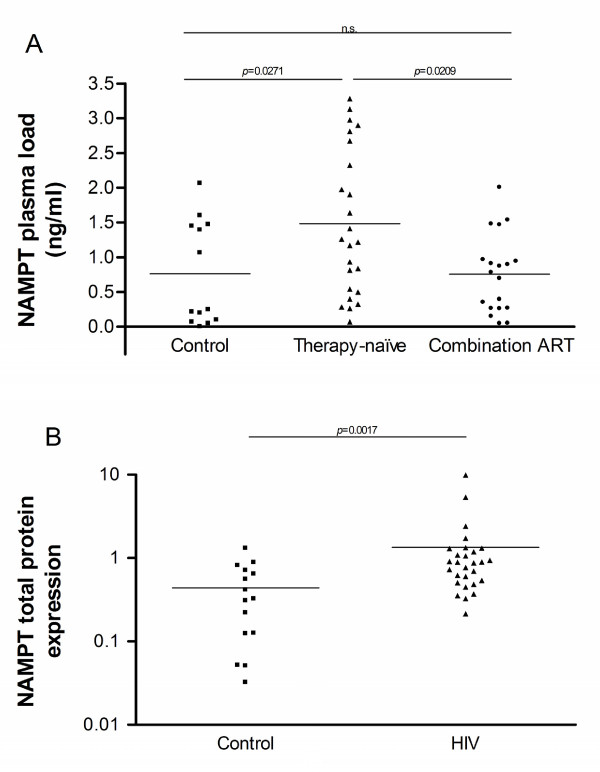
**A) Plasma levels of NAMPT versus therapy status, as assessed by ELISA (n_Control _= 13, n_Therapy-naïve _= 24, n_ART _= 19);** B) Total NAMPT protein expression in monocytes of therapy-naïve HIV patients (VL ≥ 4 log copies/ml), normalised to β-actin expression, as assessed by ECL-Western Blot (n_Control _= 15, n_Therapy-naïve _= 28). p-values calculated by nonparametric Mann-Whitney test; n.s.: not significant, ART: antiretroviral therapy.

As mentioned previously, we were particularly interested in secreted factors which are used by the monocyte/macrophage to modulate their own activity or that of other immune cells. Out of the secreted factors modulated in the therapy-naïve HIV patients, CCL2 (also known as MCP-1, Entrez GeneID 6347), NAMPT (also known as visfatin or PBEF1, Entrez GeneID 10135) and PDGFC (also known as fallotein, Entrez GeneID 56034), were found to be correlated with the viral load in therapy-naïve patients (Figure 2A-C) and for CCL2 a correlation with the CD4T count was also observed (Figure 2D). As a first step to evaluate the putative contribution of these factors to HIV infection, non-activated PBMC and MDM were pre-treated with these factors and then infected with the HIV lab strain BaL. For two out of the three factors, CCL2 and PDGFC, inconsistent effects between individuals were observed in both PBMC and MDM. The novel adipocytokine NAMPT, however, significantly inhibited HIV_BaL _infection in all donors in both cell-types (Figure 3A-B). Upon further examination, NAMPT was also capable of inhibiting the biological clones HIV_968-3 _and HIV_968-2 _[59] (Figure 3C), suggesting that the induction of this factor in monocytes during HIV infection could represent a hitherto unknown innate antiviral response. As plasma levels (Figure 4A) and total monocyte protein expression of NAMPT (Figure 4B) were also found to be elevated in HIV patients but not in patients on > 9 months combination ART, mirroring the mRNA expression levels, this factor may be of in vivo relevance during HIV infection.

### NAMPT interferes with early events of the viral life cycle

**Figure 5:**
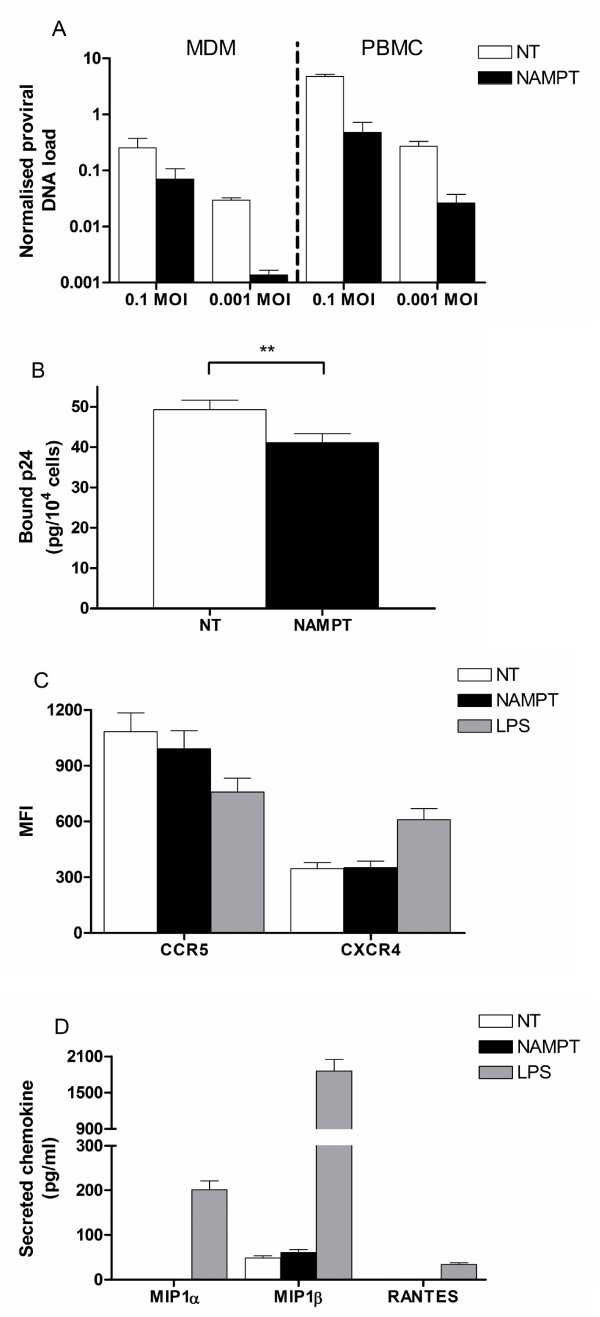
**A) Levels of integrated proviral DNA in MDM and resting PBMC (pre-)treated with NAMPT (200 ng/ml) and infected with HIV_BaL _at 0.1 and 0.001 MOI, normalised to ERV-3, as assessed by RT-qPCR;** B) viral binding to MDM, as quantified by p24 concentrations in cell lysates after 2 hours incubation and washing of the unbound virus; C) expression of CCR5 and CXCR4 on MDM treated 2 days with NAMPT (200 ng/ml) and LPS (100 ng/ml), as assessed by flow cytometry [39]; **D) secretion of the β-chemokines MIP1α, MIP1β and RANTES by MDM treated 2 days with NAMPT (200 ng/ml) and LPS (100 ng/ml), as assessed by CBA. MFI: mean fluorescence intensity**.

To evaluate at which level the effect of NAMPT may be acting, integration of proviral DNA in presence and absence of NAMPT was measured semi-quantitatively in HIV_BaL_-infected MDM and PBMC. NAMPT treatment managed to decrease the integration of proviral DNA in infected cultures (Figure 5A), suggesting that NAMPT interferes with early, pre-integration events of the viral life cycle. As viral binding and entry into the cell is a likely target of inhibitory factors, we assessed whether NAMPT could block viral interaction with the cell. While a modest reduction of HIV attachment to MDM was observed in a crude viral binding assay (Figure 5B), inhibition of infectivity was not due to modulation of CD4 (not shown) or the CCR5 coreceptor (Figure 5C) or induction of the β-chemokines MIP1α, MIP-1β and RANTES (Figure 5D), suggestive of a novel inhibitory mechanism.

## Discussion

Despite a clearly established role of monocytes and macrophages in the pathogenesis of HIV infection, the molecular mechanisms and genetic networks underpinning the myeloid dysfunctions during HIV infection have remained elusive. Using a combined approach of genome-wide microarray analysis and focused monocyte/macrophage-specific gene expression profiling, we attempted to identify genes which may contribute to the HIV-associated monocyte dysfunction *in vivo*.

Using a commercial genome-wide microarray platform we identified several biological processes which were significantly modulated by HIV infection. These processes include both previously documented pathways in the context of monocyte-HIV interactions, such as cell cycle modulation and apoptotic pathways, and processes hitherto not identified in this context by transcriptome profiling, such as lipid metabolism, protein trafficking and proteasome function. Our gene expression data are supported by previously documented *in vitro *studies in these domains [18,44].

A custom monocyte/macrophage-focused gene expression profiling platform combined with RT-qPCR validation was used to identify individual genes of interest in different areas of dysfunction in monocytes of therapy-naïve and ART-treated HIV patients. This approach was chosen for its higher cost-effectiveness and increased experimental flexibility versus a commercial microarray setup (manuscript in preparation). Our datasets reflect an aberrant immune activation of monocytes/macrophages which may be of considerable relevance for HIV pathogenesis. Specifically, we observe suppression of a cluster of factors involved in chemotaxis, suggesting an important deficiency at the level of immune cell recruitment in monocytes of HIV infected patients (table 5). Other immune response-associated genes are downregulated as well, indicative of a deficient monocyte activation state: PLA2G7, the IL1-like cytokine IL1F7 and the ion transporter SLC11A1, commonly known as Natural Resistance Associated Macrophage Protein or NRAMP1. On the other hand, the downregulation of ADORA2A and LILRB4, and the upregulation of PTGER2, IFI30, STAT1, CD83, BCL6 and NAMPT are suggestive of an activated phenotype. Our results are therefore in accordance with observations concerning a mixed phenotype of both increased and decreased pro-inflammatory features [26,27] which does not seem to be restored completely during at least the first 9 months of combination ART. A longer period of combination ART may be required to normalise this phenotype, or it may represent a true irreversible immune dysfunction in the monocyte population.

**Table 5:** Functional classification of differentially expressed genes in monocytes of therapy-naïve and HIV patients

Immune function: chemotaxis	Immune function: inactivation	Immune function: activation	Anti-apoptotic (cis)	Pro-apoptotic (trans)	Cell cycle	Protein trafficking	Transcriptional regulation	Metabolic dysregulation
*CCL23*	*ADORA2A*	*IL1F7*	*ADORA2A *[80]	*IL8 *[81]	*CCL23 *[82]	*CAPG*	*MAFF*	*CCR2 *[65]
*CCL4L1*	*LILRB4*	*PLA2G7*	**BCL6 **[83]	**HLA-DRA **[50]	*IL8 *[84]	**BCL6**	*NR0B2*	*NR0B2 *[64]
*CCR2*		*SLC11A1*	**CCL2 **[85]	**STAT1 **[86]	*NR0B2 *[47]	**MARCKS**	**BCL6**	*PLA2G7*
*CX3CR1*		**BCL6**	**CDKN1A **[87]		**BCL6 **[88]	**YWHAZ**	**CDKN1A**	**NAMPT **[66,67]
*CXCL2*		**CD83**	**NAMPT **[89]		**CCL2**		**KLF10**	
*IL8*		**IFI30**	**YWHAZ **[90]		**CDKN1A **[91]		**STAT1**	
		**NAMPT**			**HLA-DRA **[50]			
		**PTGER2**			**KLF10 **[92]			
		**STAT1**			**NAMPT**			
					**PDGFC**			
					**STAT1 **[55]			

Official Gene Symbols are shown; genes in *italics *are downregulated, genes in **boldface **are upregulated.

Most of the genes in our collection can be clustered in the functional categories identified in the genome-wide analysis. As such, our approach differs from other transcriptome analyses, in that we identify candidate genes for further analysis in a broad range of categories, rather than focussing on particular aspects of monocyte/macrophage dysfunction [26,27,60]. These clusterings are summarised in table 5.

In the context of apoptosis/DNA damage for example, we identify a cluster of genes which may contribute to the anti-apoptotic gene signature described in monocytes of HIV infected patients [15,60]. A cluster of factors which is capable of mediating apoptotic triggers in *trans *on other cells, thus contributing to lymphocyte depletion, was also identified. A similar cluster of genes possibly involved in HIV-driven cell cycle modulation [61] and multiple genes in the context of the broad biological terms protein trafficking and transcriptional regulation, reflecting the general subversion of the cellular machinery for viral purposes, were also described (table 5).

Specifically in the context of metabolic disorders, our results support the growing notion that metabolic dysregulation in the context of HIV infection is probably not limited to the phase under ART, but is a pre-existing condition, manifesting sub-clinically during therapy-naïve HIV infection [62,63]. We have indeed identified a set of genes dysregulated by HIV itself which may be capable of modulating lipid metabolism. Downregulation of the nuclear factor NR0B2 can via several intermediaries increase the catabolism of cholesterol [64]. Downregulation of the acetylhydrolase PLA2G7 may result in an increased risk for atherosclerosis, though the role of this enzyme in this field is still contentious. The decreased expression of CCR2 [65] and increased expression of NAMPT [66,67] may impact on atherosclerotic lesion formation. In the context of ART-associated complications, finally, we have identified several genes which are reported to be linked with lipodystrophy and/or the metabolic syndrome as modulated under ART (CAPZA1 [68], CCL2 [69], GAS6 [70], NAMPT [71,72], STAT1 [73]), suggesting that the monocyte population may contribute to the development of ART-associated metabolic disorders through these factors.

Additionally, the genes identified in our study may of course play unexpected roles in other manifestations of monocyte/macrophage dysfunction. Because of the interesting properties of secreted factors, which represent the means by which monocytes/macrophages can mediate many of their effects in autocrine or paracrine fashion [29,33,34], we focused on three factors which we identified as differential and which showed an association with the viral load in therapy-naïve HIV patients. For two factors, CCL2 and PDGFC, no consistent effects were observed on HIV infectivity in PBMC and MDM. For the novel adipocytokine NAMPT/visfatin, however, an inhibitory effect was observed on HIV infection in both cell types for both a lab-attenuated strain and two biological clones. NAMPT may thus represent an (interferon-induced) antiviral factor which is elicited in response to higher levels of circulating virus. Indeed, in silico profiling of NAMPT expression using the web application Genevestigator [74] suggests that it is upregulated in multiple models of viral infection, including infections with CMV, measles virus, herpes simplex virus, rotavirus and adenoviruses.

NAMPT appears to act on early events of the viral life cycle, as the integration of proviral DNA is abrogated by NAMPT treatment. A plausible mechanism for the inhibitory activity of NAMPT would therefore be blocking of viral binding to the cell; indeed, binding of HIV is reduced in the presence of NAMPT. However, the observed modest reduction may suggest that viral binding is not the only or even the most important factor in NAMPT activity. Additionally neither modulation of (co-)receptor expression or induction of β-chemokine secretion by visfatin could be demonstrated. Possible other aspects of viral binding and/or post-entry/pre-integration effects remain to be evaluated. In this regard, the role of NAMPT in TNF regulation through its function in cellular energy metabolism [75] seems a promising research avenue.

Finally, we analysed the gene expression patterns in monocytes from patients who developed a hypersensitivity reaction to abacavir, a severe and potentially lethal adverse reaction to the drug. Compelling evidence for the involvement of antigen presenting cells in general and monocytes in particular in the development of the abacavir hypersensitivity reaction was recently published [76]. Six genes were identified as differentially expressed at baseline between patients who developed the hypersensitivity reaction and patients who were initiated on the same regimen and had a beneficial response. These genes may provide a first basis for investigations aimed at the identifying bio-markers for the development of the abacavir hypersensitivity reaction. This could be especially useful in populations where the HLA-B*5701 genotypic screening lacks predictive value [77]. Furthermore, they may play an important role in the molecular mechanisms underlying this detrimental form of immunopathology. Two genes in particular may be of functional interest: upregulation of CYP2C19, a member of the cytochrome P450 monooxygenase family with a reputation for antiretroviral drug interactions [78], may lead to a higher availability of abacavir metabolites, which may in turn trigger the hypersensitivity reaction. Higher expression of the inflammatory chemokine CCL1, on the other hand, is associated with so-called M2b or type 2 alternative monocyte activation [79], and may thus be indicative of a predisposition to allergic/hypersensitivity reactions.

An unexplored aspect of this study is an inherent limitation to all transcriptome analyses. In our setup, it cannot be ascertained to what extent the differential genes which we identify are dysregulated to a limited degree in the complete monocyte population or to a high degree in a limited monocyte subpopulation (such as only the fraction of infected monocytes in the blood). However, considering the limited number of infected monocytes in the peripheral blood, it is likely that the changes in gene expression which we record here are the result of external factors on the complete monocyte population (such as circulating viral antigens or secreted host-derived factors) rather than direct infection of individual monocytes.

In this study of *ex vivo *monocytes from HIV patients, we have identified several key areas of cellular dysfunction, and we have pinpointed multiple genes associated with both HIV infection and antiretroviral therapy in these key areas. These genes represent an interesting population for further in-depth functional studies concerning their role in HIV pathogenesis. A first candidate for further functional analysis could be the factor NAMPT/visfatin, which shows a strong correlation with the viral load in patients, and which seems to mediate an inhibitory effect for HIV infection in both PBMC and MDM.

## Competing interests

The authors declare that they have no competing interests.

## Authors' contributions

RVB designed and performed research and drafted the manuscript, JG, PDB, GV and GR designed and discussed research, EF and EV consulted patients and provided biological samples and clinical data, TB, EH, HTTT and YG designed and performed research. All authors read and approved the final manuscript.

## Supplementary Material

Additional file 1 **Gene collection represented on the custom Macrophage Activation State array platform**. All genes for which amplified cDNA probes were printed on the custom Macrophage Activation State array platform are represented; Probe ID: a unique identifier for each set of gene specific primers used to generate cDNA probes; OGS: Official Gene Symbol (Entrez Gene); Entrez ID: official Entrez Gene identifier; Transcript variants: recognition of individual transcript variants by the cDNA probe, if applicable (All: global probe hybridising with all known transcript variants; N.A.: no transcript variants known at time of production). Click here for file

Additional file 2 **Gene specific primer sets for real-time semi-quantitative PCR**. Sequences of gene specific primer sets used in real-time semi-quantitative PCR are shown; OGS: Official Gene Symbol (Entrez Gene); Entrez ID: official Entrez Gene identifier. Click here for file

Additional file 3 **Genes expressed differentially in monocytes from HIV patients versus healthy controls, as assessed by CodeLink HWG microarray analysis**. Samples analysed by CodeLink HWG microarray were grouped according to HIV serostatus and were analysed for differential gene expression. Standard CodeLink identifiers are shown (CodeLink unique probe name, NCBI accession number and NID, Entrez Gene ID (LocusLink) and UniGene ID); p-val_uncorrected: p-value of Student's t test (astringent); p-val_corrected: p-value of Benjamini-Hochberg corrected Student's t test (stringent); Fold_change: fold change between the means of the two groups (HIV/control). Click here for file

Additional file 4 **Differential gene expression in patients with a beneficial reaction versus a hypersensitivity reaction to abacavir**. Gene expression values as assessed by the Macrophage Activation State array platform in monocytes of HIV patients who develop the hypersensitivity reaction to abacavir versus patients with a beneficial response to the same therapy regimen; gene expression assessed at baseline before initiation of therapy. Gene expression was mean centred. Official Gene Symbols are shown, Entrez Gene identification codes are mentioned in parenthesis. Click here for file
