# Supplementary figures and images for: Transcriptome analysis of monocyte-HIV interactions
Source: Retrovirology. 2010 Jun 14;7:53. doi: 10.1186/1742-4690-7-53 (PMC2900222; doi:10.1186/1742-4690-7-53)

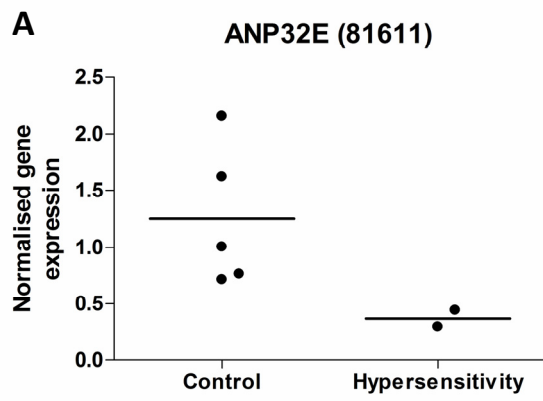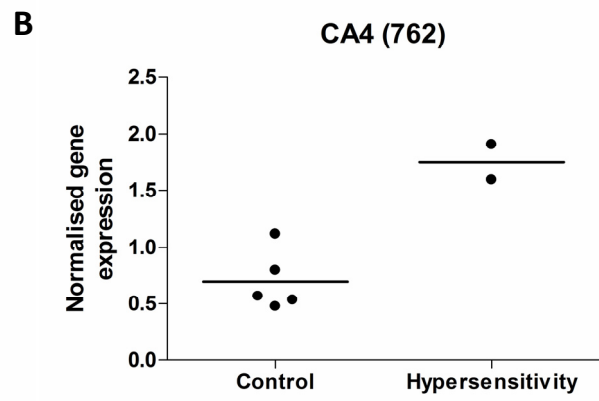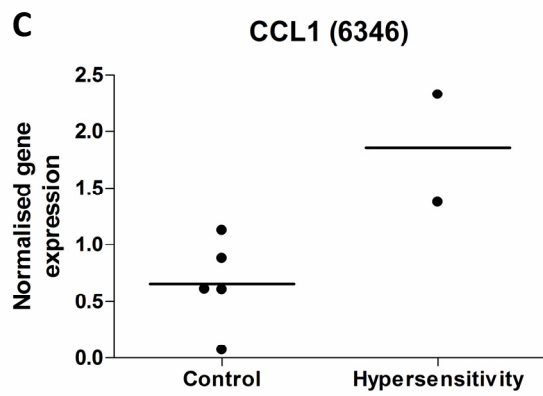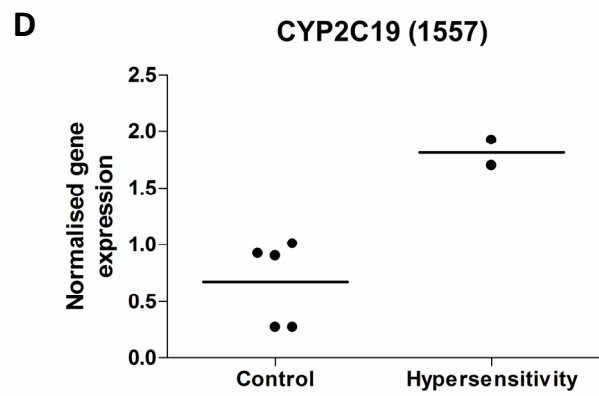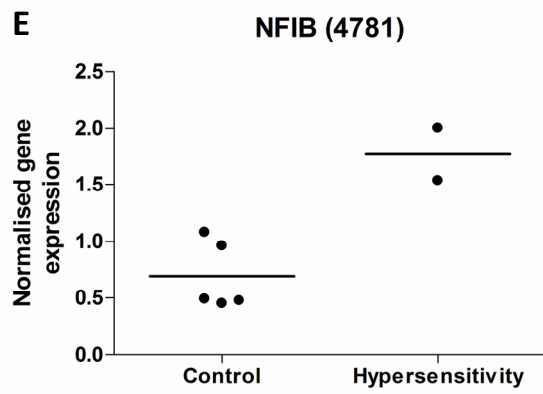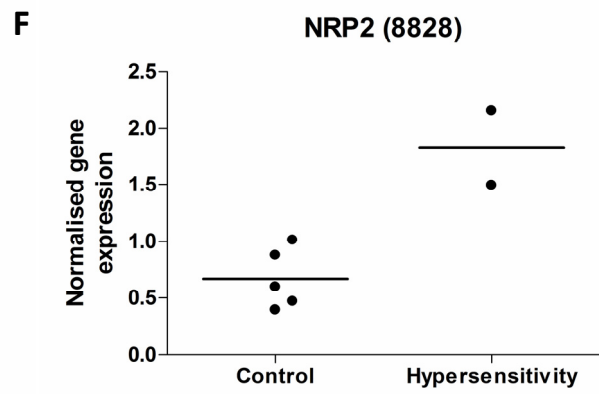

Supplement: Additional file 4 — Differential gene expression in patients with a beneficial reaction versus a hypersensitivity reaction to abacavir. Gene expression values as assessed by the Macrophage Activation State array platform in monocytes of HIV patients who develop the hypersensitivity reaction to abacavir versus patients with a beneficial response to the same therapy regimen; gene expression assessed at baseline before initiation of therapy. Gene expression was mean centred. Official Gene Symbols are shown, Entrez Gene identification codes are mentioned in parenthesis. [file 1742-4690-7-53-S4.PDF]
